# Investigating oral microbiome dynamics in chronic kidney disease and post-transplantation in continuous culture

**DOI:** 10.1128/spectrum.00598-24

**Published:** 2024-10-09

**Authors:** Paul M. Campbell, Thomas Willmott, Angela Summers, Christopher G. Knight, Gavin J. Humphreys, Joanne E. Konkel, Titus Augustine, Andrew J. McBain

**Affiliations:** 1 School of Health Sciences, Faculty of Biology, Medicine and Health, The University of Manchester, Manchester, United Kingdom; 2 Department of Renal and Pancreatic Transplantation, Manchester Academic Health Science Centre, Manchester University Hospitals NHS Foundation Trust, Manchester, United Kingdom; 3 School of Natural Sciences, Faculty of Science and Engineering, The University of Manchester, Manchester, United Kingdom; 4 School of Biological Sciences, Faculty of Biology, Medicine and Health, The University of Manchester, Manchester, United Kingdom; The Ohio State University College of Dentistry, Columbus, Ohio, USA

**Keywords:** urea, oral microbiome, kidney transplantation, chronic kidney disease

## Abstract

The oral microbiome is influenced by environmental factors in chronic kidney disease and following kidney transplantation affecting microbial composition, which may have implications for health and recovery. A major driver of oral microbiome perturbation is the accumulation of urea in saliva. We have modelled increased salivary urea concentrations associated with CKD and subsequent reductions that may occur post-transplantation. Oral microbiota were established in constant-depth film fermenters by inoculation with saliva. Duplicate validation runs were maintained with artificial saliva with baseline urea concentrations (0.205 mg/mL) for 21 days. Triplicate treatment runs were then done with baseline urea for 10 days (healthy phase) before urea was increased for 10 days to reflect CKD concentrations (0.92 mg/mL) (CKD phase). This was followed by reversion to baseline urea concentrations (post-transplant phase). Biofilms in primary validation runs reached dynamic stability within 5 days according to viable counting. DNA sequence data indicated minimal taxonomic variation over time and between low and high urea treatments despite background noise indicating changes in bacteria belonging to the family Gemellaceae and the genera TG5 and *Leptotrichia*. Significant differences in alpha and beta diversity occurred between low and high urea states but not following reversion to a low urea environment. Increased abundance of the TG5 was detected in late model phases, despite apparent count stability, and independent of changes in urea concentrations.

**IMPORTANCE:**

This study investigates dynamic changes in the oral microbiome associated with changes in salivary urea concentration, an important factor in chronic kidney disease (CKD). The *in vitro* system modeled increased urea concentrations and subsequent reductions post-transplantation. The study provides insight into the oral microbial shifts during different simulated clinical phases. Understanding these dynamics is crucial for advancing our comprehension of CKD-associated oral microbiome variations and their potential impact on patient well-being and recovery.

## INTRODUCTION

The oral microbiota changes compositionally following kidney transplantation (1). Studies have proposed that changes in the oral microbiota are linked to post-operative outcomes, including increased gingival overgrowth and *Candida* infection (2, 3). Using high-throughput sequencing techniques, two studies have reported increases in the abundance of opportunistic pathogens and decreased community diversity (4, 5). This is of particular potential concern, given the susceptibility of immunocompromised recipients to severe infections, which occur at an incidence of 25.5% within the first-year post-transplantation (6). Furthermore, the oral microbiome has been proposed as a reservoir for extra-oral colonization, which has implications beyond driving severe infections as this could drive inflammation (7) and which may increase the risk of allograft rejection.

Prior to, and during, kidney transplantation, the oral microbiome is subjected to several stressors, which could potentially drive observed changes in diversity and composition. These factors could be those associated with surgery and extended hospital stay. Broadly, chronic kidney disease (CKD), for which kidney transplantation is the gold-standard therapy, is associated with oral microbiome change. Differences in the oral microbiome of CKD patients include enrichment of *Neisseria* and depletion of *Veillonella* compared with those without CKD (8). A major CKD-associated factor which might play a role in driving microbial change is the availability of uremic toxins (9). As renal function reduces through the different stages of CKD, the body’s ability to excrete toxins and waste and metabolize vitamin D is also reduced (10–12). As uremic toxins begin to accumulate, they begin to interact with the immune system and potentially drive multiple immune system disturbances seen in uremic end-stage renal disease (ESRD) patients (13).

As kidney function declines, the metabolism of uremic toxins, urea and creatinine, is reduced, and their serum concentration increases (14, 15). This rise in serum concentrations is correlated with an increase in salivary concentrations (16), with CKD patients demonstrating higher salivary creatinine and urea levels than healthy controls (16). The effect of “above normal” levels of salivary urea is understudied in the literature. The resulting increase in localized environmental pH could have cascading effects on the network of microbiota in the oral cavity. Increased pH could have a differential effect on oral bacteria with different pH optima (1, 17–20). For instance, oral nitrate reduction to nitrite has been suggested to be key in systemic cardiovascular regulation (21–23) and is higher at a lower pH (24, 25). Ammonia production from arginine metabolism increases pH in a self-regulatory mechanism (26, 27). Therefore, the complex relationship between the oral microbiome, its metabolism, and host systemic health remains largely uncharacterized in CKD.

The consequences of a more alkaline oral environment could go beyond the differences in microbial composition observed between CKD patients and healthy controls (8). A more basic environment could offer greater protection to enamel against acidification and demineralization (28, 29). The low prevalence of tooth decay in CKD is a long-documented phenomenon (30, 31). An investigation into the properties of the saliva and dental plaque of CKD patients found that plaque pH correlated directly with salivary urea nitrogen [often measured in blood or saliva, an indication of kidney function (32, 33)] concentration. Significantly, the same study also concluded that following a challenge with carbohydrate exposure, the minimum pH of CKD subjects did not reach cariogenic levels (while non-CKD subjects did) (31). Declining kidney function, therefore, might begin a process whereby increased salivary urea concentration drives microbial production of ammonia, which raises environmental pH, alters oral microbial profiles, and has consequences on oral health. Once this system has been established in the CKD host, the removal of chronic kidney disease by kidney transplantation is likely to cause further perturbation. It is unknown whether the return to normal salivary urea concentration causes the reversal of the above process, lowering pH and leaving the host more vulnerable to cariogenesis.

There are numerous concomitant factors which could influence the oral microbiome during kidney transplantation. Studying this effect in human transplant subjects is complicated by confounders, such as perioperative antibiotics, the introduction of immunosuppressive agents, and the effects that variation in oral pH (34, 35) and uremia (13) can have on the immune system. To study this effect in isolation, therefore, an *in vitro* modeling approach was used in the present study to understand how high salivary urea concentration can affect the composition and diversity of the CKD oral microbiome.

## MATERIALS AND METHODS

### Growth media

Bacterial isolates were grown on Wilkins Chalgren agar (WCA) (Scientific Laboratory Supplies, UK) and incubated overnight at 37°C under aerobic conditions. To grow liquid cultures, a single colony from a WCA plate was added to 10 mL of Wilkins Chalgren broth and grown aerobically overnight at 37°C with 200 rpm shaking.

### Maintenance of oral microcosms

Oral microcosms were maintained in constant-depth film fermenters (CDFFs) as previously described (36–40). As per Ledder and Mistry (36), the temperature was maintained at 36°C by housing the CDFFs within perspex incubation chambers (Stuart Scientific, Redhill, Surrey, UK). The CDFF plugs were formed of polytetrafluoroethylene (PTFE). While hydroxyapatite has been commonly used in the past for plug surfaces in similar studies, the long-term growth of acidogenic plaques could risk dissolution (36); hence, PTFE was preferred. The PTFE plugs were set to a depth of 200 µm with a turntable rotor speed of 3 rpm. For artificial saliva medium, a semidefined McBain medium (pH 7) (41, 42) containing 2.5 g/L mucin, 2 g/L Bacto peptone, 2 g/L Trypticase peptone, 1 g/L yeast extract, 0.35 g/L NaCl, 0.2 g/L KCl, 0.2 g/L CaCl_2_, 1 mg/mL hemin, and 2 mg/L vitamin K1 was added to the fermenter at 8 mL/h by a peristaltic pump (Minipuls 3; Gilson). Before inoculation, the PTFE plug surfaces and CDFF were conditioned for 24 hours with McBain medium at 36°C. The fermenters were inoculated with fresh saliva from a single healthy individual donor (age = 28 years) on two separate occasions (4.0 ± 0.5 mL/fermenter/inoculation) 10 hours apart. Sampling was performed every 48 hours; to avoid sampling immature plaques [per Ledder and Mistry (36)], pans were numbered and sampled sequentially. Samples were processed immediately for bacteriological analysis or stored at −80°C before extraction for high-throughput sequencing.

### Validation of medium with the addition of urea

To validate the use of McBain medium with the addition of urea, two CDFFs were established with McBain medium with the addition of urea at the concentration found in the saliva of healthy controls by Lasisi et al. (16) (0.205 mg/mL). Previous studies have shown that dental biofilm communities within CDFFs reached dynamic steady states between 2 and 7 days (36, 41), as evidenced by viable count data. To validate the establishment of dynamic stable communities of oral biofilms in McBain medium with additional urea, communities of dental biofilms were sampled every 48 hours from two CDFFs for 21 days post-inoculation.

### Oral microcosm modeling of chronic kidney disease and post-transplant states

Following the establishment of a stable population size in oral microcosms with low urea in two “validation” models, three “treatment” models were established in CDFFs to challenge these environments with CKD (high urea) and post-transplant (low urea) states. Based on a study by Lasisi et al. (16), median salivary urea levels of CKD patients (92.00 mg/dL) were 4.5 times higher than healthy controls (20.50 mg/dL). For the three “treatment” models, CDFFs were conditioned with low-urea McBain medium (urea concentration 0.205 mg/mL) at 36°C for 24 hours before inoculation. CDFFs were sampled 24 hours after inoculation and then every 48 hours until day 31. After 10 days post-inoculation (hereafter referred to as the “healthy phase”), the flow of the low-urea McBain medium was stopped, and high-urea McBain medium (0.92 mg/mL) was commenced at the same flow rate (8 mL/h) for 10 days (hereafter referred to as the “CKD phase”). Following 10 days at the CKD phase, the flow of high-urea McBain medium (0.92 mg/mL) was stopped, and drip feeding with fresh low-urea McBain medium was commenced for 10 days (post-transplant phase).

### Bacteriological analysis of CDFF samples

Five plugs were derived from each aseptic sampling (at each time point). Four plugs were chosen and split into pairs (at random). Pairs of sample plugs were each placed into separate universal containers (becoming replicates) and each vortexed for 1 min with 2-mm glass beads in 5 mL phosphate-buffered saline (PBS). Appropriate dilutions (0.2 mL) to target the production of plates containing 30–300 colonies were produced, and replicates were then serially diluted in (1.8 mL) PBS and plated in duplicate on selective and non-selective media. Where plates became contaminated or counts were below 30, plates with the highest available colony count were selected. For total aerobe counts, 0.2 mL of these dilutions was plated onto WCA and placed into an aerobic incubator at 37°C for 4 days. For total anaerobe counts, dilutions were plated onto WCA and placed into an anaerobic chamber (Don Whitley Scientific, Shipley, UK), with an atmosphere of 10% H_2_, 10% CO_2_, and 80% N_2_ for 4 days. For total counts of *Streptococcus* species, dilutions were plated onto the streptococci-specialist (Mitis Salivarius) agar (Merck Life Science, UK). Colony counts were performed after 4 days.

### High-throughput sequencing of CDFF samples

After extraction of sample plugs and vortexing with glass beads in PBS, 2 mL of the PBS medium was stored at −80°C for high-throughput sequencing. From the stored CDFF oral microcosm samples, 250 µL was extracted via the DNeasy Power Soil Pro Kit (Qiagen, Manchester, UK), according to the manufacturer’s instructions. Overall, 70 samples from the CDFFs were extracted; for each batch of extractions, a positive control (DNA from a culture isolate in 250 µL PBS) and an extraction negative control (250 µL PBS only) were included. For each batch of PCRs performed, a PCR-positive (a previously tested extraction of *Lactobacillus plantarum*) and PCR-negative [PCR-grade water (Qiagen, UK)] control were also included. Following extraction, the DNA was amplified by PCR using the 515F (5′-TCGTCGGCAGCGTCAGATGTGTATAAGAGACAGGTGYCAGCMGCCGCGGTAA-3′) and 806R (5′-GTCTCGTGGGCTCGGAGATGTGTATAAGAGACAGGGACTACNVGGGTWTCTAAT-3′) primers to amplify the variable region 4 (V4) of the 16S rRNA gene (43–45). Extracted DNA was added at a volume of 2.5 µL, followed by 5 µL of 515F primer, 5 µL of 806R primer, and 12.5 µL of KAPA HiFi HotStart ReadyMix (Roche, London, UK) with a final PCR reaction volume of 25 µL. Following a denaturing step at 95°C for 3 min, PCR was performed with 25 cycles of 95°C for 30 seconds, 55°C for 30 seconds, and 72°C for 30 seconds, followed by an elongation step of 72°C for 5 min. Amplification was confirmed with gel electrophoresis. After positive bands were confirmed (or absent, in the case of negative controls), samples were submitted to Deep Seq Next Generation Sequencing Facility at the University of Nottingham (Nottingham, UK) for sequencing via Illumina MiSeq.

### High-throughput sequence data processing

Paired-end sequence data were imported into QIIME2 version 2022.2 (46) via the Casava 1.8 paired-end demultiplexed fastq format. From demultiplexed sequences, before denoising, there were 4,118,143 paired forward and reverse reads (median per sample: 49,148; range: 65–77,216). Since DADA2 is a denoiser stated for use with Illumina-generated data (47), this denoiser was preferred to the Deblur option (48). The amplicon sequence variants (ASVs) generated by DADA2 were aligned using mafft (49), and a phylogeny was constructed using FastTree2 (50). After denoising (including internal quality control and filtering of phiX reads and chimeric sequences), 3,350,577 reads remained with 549 ASVs identified. After rarefaction (subsampling without replacement) to an even depth, alpha and beta diversity metrics were estimated by q2 diversity (46). A rarefaction depth of 29,919 was chosen for diversity analyses [validation run (V) V1: 11, V2: 12; treatment run (T) T1: 16, T2: 14, T3: 15]. Alpha diversity was measured via Shannon’s diversity index (51), Pielou’s evenness (52), Faith’s phylogenetic diversity [PD; (53)], and the observed ASVs (54, 55). Significant differences in alpha diversity metrics between groups were tested using unpaired *t*-test or one-way analysis of variance (ANOVA) tests (and, where significant, with Tukey’s multiple comparison test) within GraphPad Prism 9 (GraphPad Software, California, US) and plotted using the same software. For beta diversity analysis, four metrics applied to each data set before analysis were Jaccard (56), Bray-Curtis (57), unweighted UniFrac, and weighted UniFrac (58, 59). The clustering of beta diversity metrics between these groups was tested via permutational multivariate ANOVA (PERMANOVA; 999 permutations) within QIIME2 (60), and the principal coordinates analysis (PCoA) plots were generated in Rstudio (61) using the qiime2R (62), phyloseq (63), and tidyverse (64) packages. Taxonomy was assigned using the q2-feature-classifier (65) classify-sklearn Naïve Bayes taxonomic classifier against the Greengenes 13_8 97% OTU reference sequences (66). Stacked taxonomic bar plots were produced using the ggplot2 package (67) in Rstudio (61). Following the recommendations of Nearing et al. (68), three differential abundance testers were selected to look for differentially abundant taxa. Before differential abundance testing, the sample from day 27 of treatment run 3 (T3) was removed due to low ASV count (27) and the presence of only ASVs belonging to *V. dispar* at 100% relative abundance. Differential abundance testing was performed within QIIME2 via ANCOM (69) and ALDEx2 (70) using the q2-aldex2 plugin (71, 72). The third differential abundance tester was the DESeq2 R package (73), which was chosen due to its high sensitivity in small data sets (74).

## RESULTS

### Validations runs

To test the effects of urea on the oral microbiota, we used microcosms, seeded from saliva, cultured for a period of weeks, with regular sampling, in CDFFs. We started with runs to validate the use of CDFFs with McBain medium and urea at the level of saliva from healthy individuals before moving on to treatment runs where urea levels were manipulated. Compared to the saliva donor, validation runs did not differ by alpha diversity (*P* = 0.13) but did by beta diversity (weighted UniFrac) (*P* = 0.012) (data not shown).

### Dynamic stability in validation runs by total colony counts

**Fig 1:**
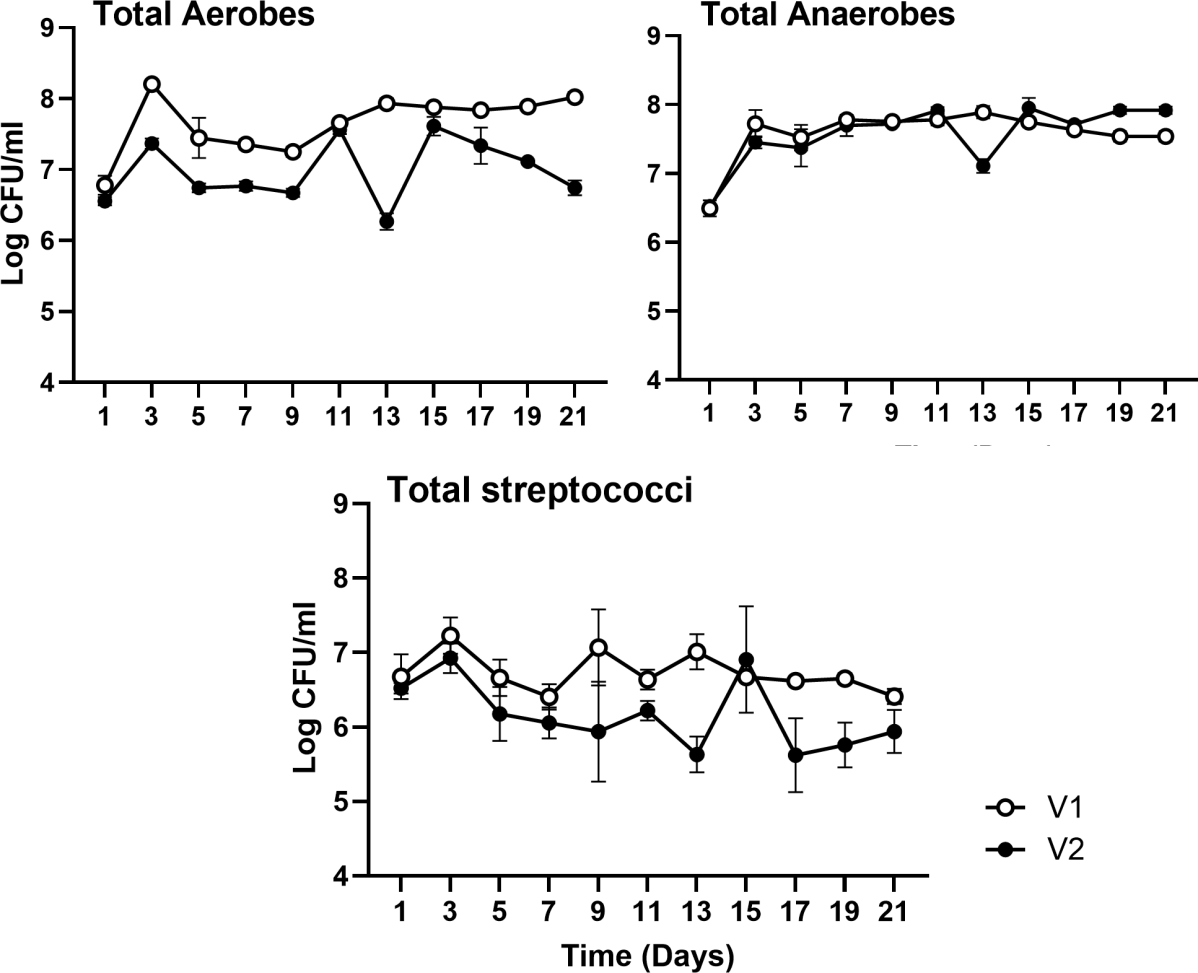
Population dynamics in oral biofilm microcosms drip fed McBain medium with the addition of urea at the median concentration found in the saliva of healthy controls (0.205 mg/mL). Open and closed symbols differentiate duplicates (open symbol, validation V1; closed symbol, validation V2). Data are means ± standard deviation.

Validation CDFF models were run in duplicate for 21 days with no change in conditions to determine model stability. Dynamic stability of oral microcosm biofilms was achieved in both validation (V1 and V2) runs around 5 days after inoculation. This is evidenced by colony counts (Fig. 1), which showed the maintenance of stable colony counts for total aerobes, anaerobes, and streptococci from day 5 until day 21 after inoculation. The validation runs established oral microcosm communities with similar mean log_10_ colony-forming units per milliliter (log CFU/mL) of total aerobes.

### Dynamic stability in treatment runs by total colony counts

**Fig 2:**
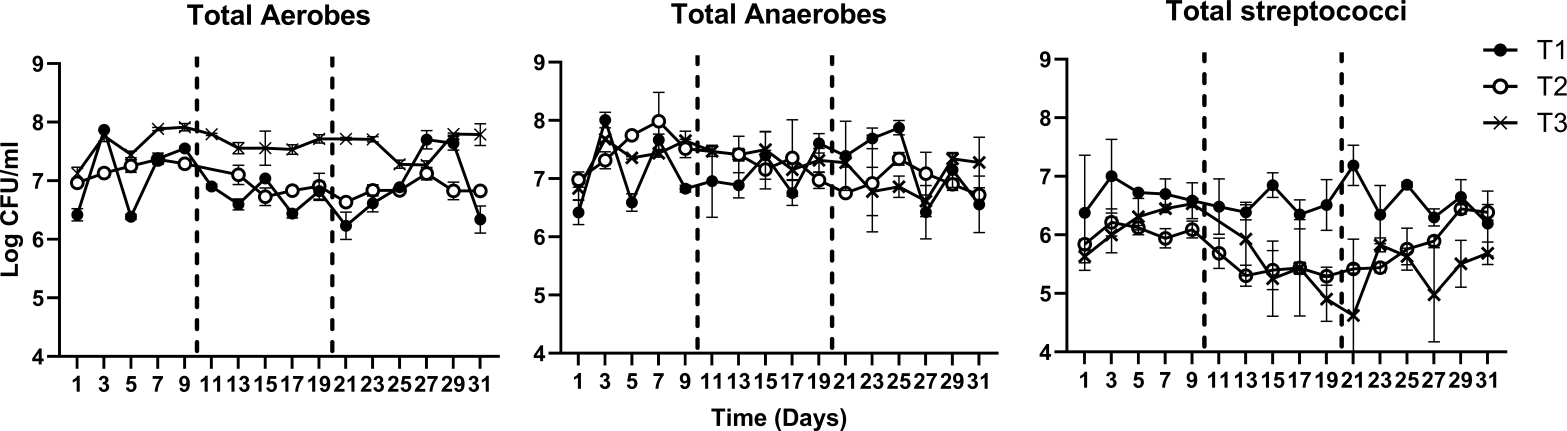
Population dynamics in oral biofilm microcosms drip fed McBain medium with the addition of urea at the median concentration found in the saliva of healthy controls (0.205 mg/mL) for 10 days, followed by urea at the median concentration found in the saliva of chronic kidney disease patients (0.92 mg/mL), followed by a further 11 days at a median concentration of healthy controls. Open, closed, and cross symbols differentiate triplicates (closed symbol, treatment run T1; open symbol, treatment run T2; cross symbol, treatment run T3). Data are means ± standard deviation (*n* = 3 model runs).

Colony counts indicated that oral microcosm communities were quickly established in dynamic stability (at a stable population size), and this was maintained for 31 days following inoculation (Fig. 2). For treatment run 1 (T1), the mean log CFU colony count (per milliliter) of total aerobes was 6.93 (range: 5.23–7.87).

### Alpha diversity of validation runs in early (days 1–10) and late (days 11–21) phase

**Fig 3:**
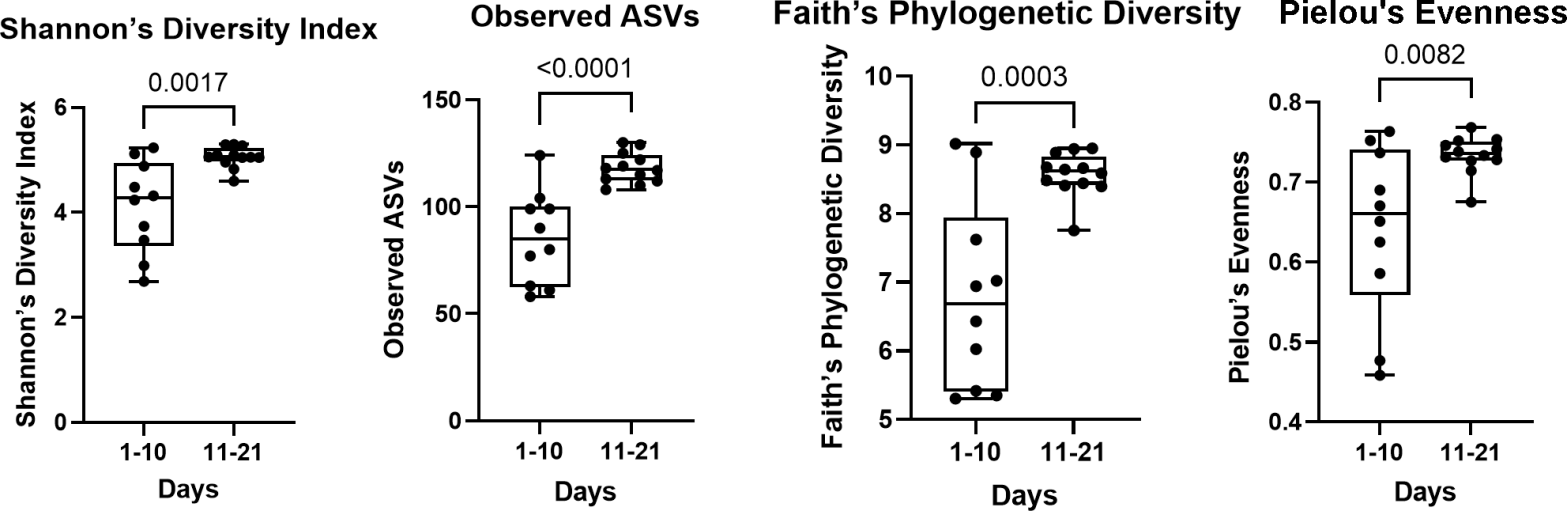
The alpha diversity of 68 samples taken from 2 CDFF validation runs (V1 and V2), comparing the early (days 1–10) and late (days 11–21) phases via four metrics (Shannon’s diversity index, observed ASVs, Faith’s phylogenetic diversity, and Pielou’s evenness). The significance of statistical (*t*-test) comparisons between groups is indicated above brackets.

The difference in alpha diversity between the first 10 days post-inoculation and the last 11 days post-inoculation in validation runs was measured using four metrics (Fig. 3). Comparing these groups via *t*-test, the “late” phase of the CDFF run was found to have higher alpha diversity via all four metrics.

### Beta diversity of validation runs in early (days 1–10) and late (days 11–12) phase

**Fig 4:**
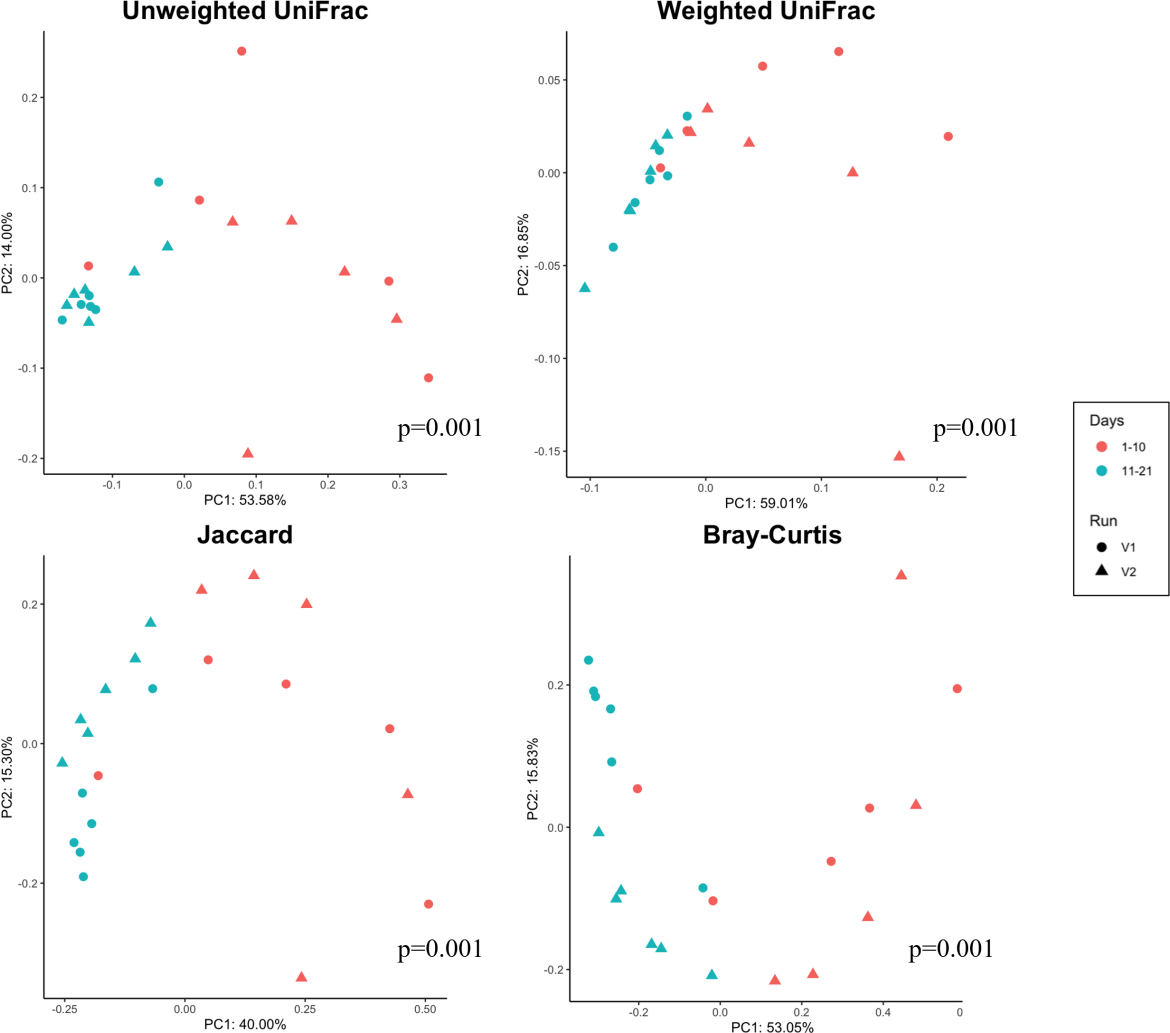
Beta diversity analysis of samples from validation CDFF runs (V1, circle symbols; V2, triangle symbols) measured via four metrics (Jaccard, Bray-Curtis, unweighted UniFrac, and weighted UniFrac) for early (days 1–10) and late (11–21) phases, shown in red and blue, respectively. Significance, as detected via PERMANOVA testing, is indicated in the bottom right of each.

Beta diversity was calculated using principal coordinates analysis with four metrics Jaccard, Bray-Curtis, unweighted UniFrac, and weighted UniFrac. Plotting these data (Fig. 4) revealed differential clustering patterns in all four metrics between samples taken from the early and late phases. This was confirmed statistically by permutational multivariate ANOVA, which found significant differences between early and late phases using all four metrics (*P* = 0.001). In all four cases, the separation between early and late is largely along the first principal component and that the late samples have a much narrower distribution on that axis than the early samples, suggesting a degree of stabilization over time.

### Relative abundance of genera for validation runs (V1 and V2)

**Fig 5:**
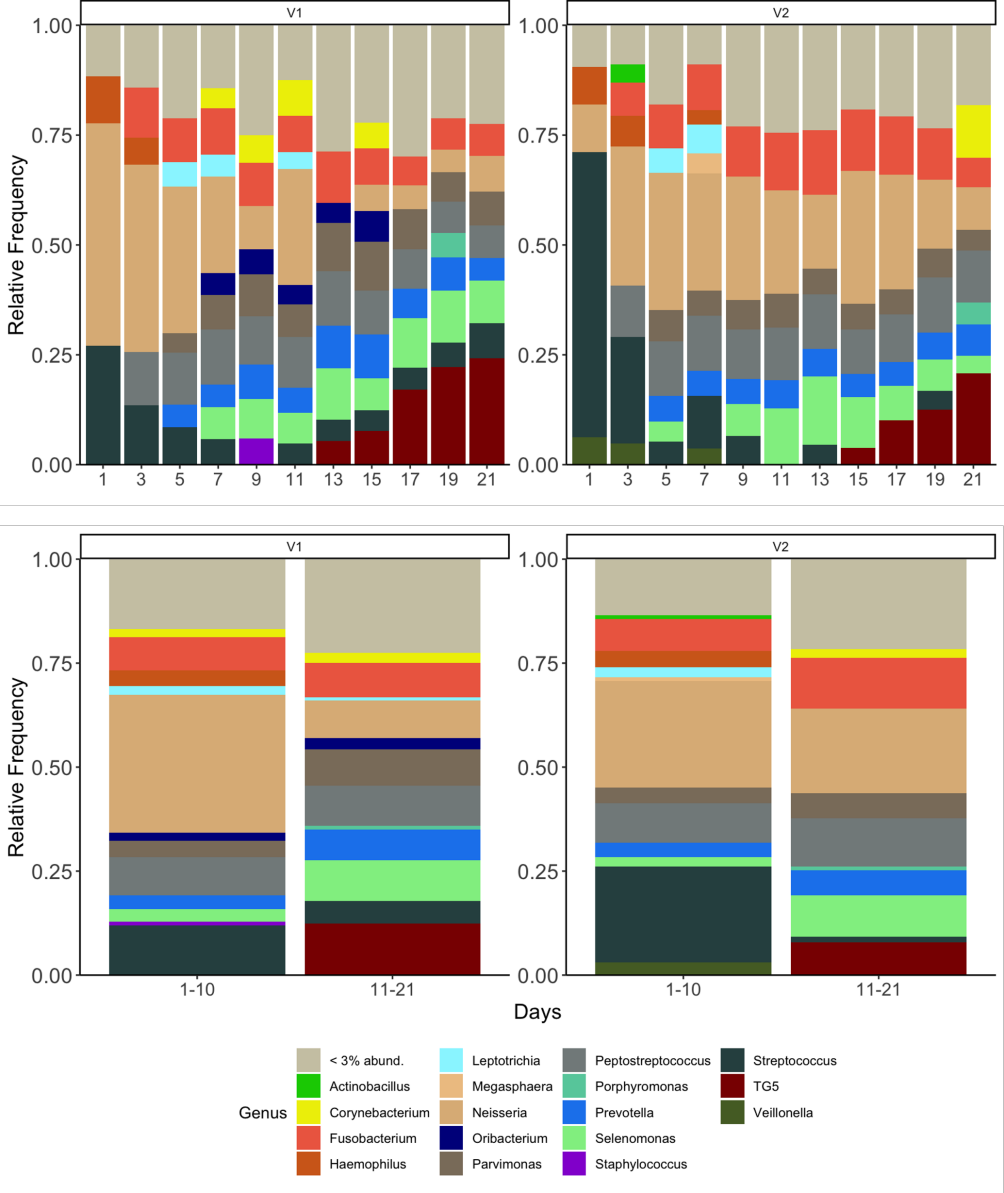
(Top) Microbiome profiles of oral microcosm samples collected from CDFF validation runs (V1 and V2). The stacked bar chart shows the mean proportion of genera (relative abundance) in each sample from each validation run. (Bottom) Mean relative abundance of genera from each validation run in early (days 1–10) and late (days 11–12) phase. Each genus is represented by a different color (legend below). Genera found at relative abundance below 3% are grouped together (gray).

The two validation runs developed established microbiome profiles with *Streptococcus*, *Peptostreptococcus*, *Prevotella,* and *Neisseria* among the genera above 3% abundance in phases in both runs (Fig. 5). The 10 genera with the highest relative abundances (of the 2 runs combined) are shown for each phase in Table 1 and include *Neisseria*, *Streptococcus*, *Peptostreptococcus*, *Fusobacterium*, *Selenomonas*, *Parvimonas*, *Prevotella*, TG5, *Leptotrichia,* and *Oribacterium*. In the late phase, a gradual increase in TG5 is apparent in both runs beginning at days 13 (V1) and 15 (V2) (respectively). A decrease in the relative abundance of *Neisseria* and *Streptococcus* is also apparent in the late phase.

**TABLE 1:** The 10 defined genera with the highest combined (model runs) relative abundance^*a*^

Genera	Days 1–10 (%)	Days 11–21 (%)
*Neisseria*	26.49	11.98
*Streptococcus*	15.12	3.09
*Peptostreptococcus*	8.69	8.23
*Fusobacterium*	7.27	8.00
*Selenomonas*	2.53	7.50
*Parvimonas*	3.69	5.55
*Prevotella*	3.37	5.12
TG5	0.18	7.23
*Leptotrichia*	2.92	1.53
*Oribacterium*	1.83	2.61

^*a*^ Relative abundance of each genus in the early (days 1–10) and late (days 11–21) phases is shown.

### Treatment runs

To test the effects of kidney transplantation on the oral microbiota, CDFF runs were in three consecutive phases: healthy (low urea), CKD (high urea), and post-transplant (low urea), with each phase lasting 10 days.

### Alpha diversity of treatment runs in healthy, CKD, and post-transplant phases

**Fig 6:**
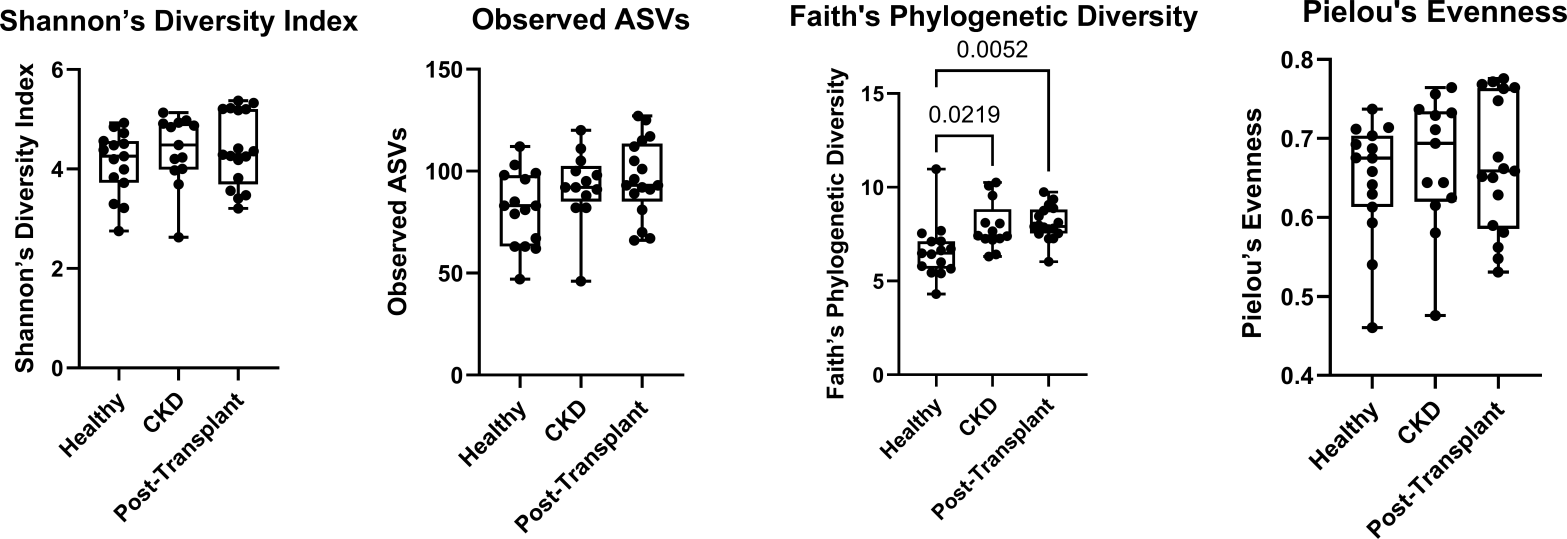
The alpha diversity of samples taken from the CDFF “treatment” runs (T1, T2, and T3), comparing the “healthy” (days 1–10), CKD (days 11–20), and “post-transplant” (days 21–31) phases via four metrics (Shannon’s diversity index, observed ASVs, Faith’s phylogenetic diversity, and Pielou’s evenness). The significance of statistical comparisons between groups is indicated above brackets (where *P* < 0.05).

The difference in alpha diversity between the three phases of feeding with (low or high urea) artificial saliva “treatment” runs was measured using four metrics (Fig. 6). Comparing these groups via one-way ANOVA, no differences were found between phases using Shannon’s diversity index (*P* = 0.4641), observed ASVs (*P* = 0.0711), or Pielou’s evenness (*P* = 0.7551). A one-way ANOVA, performed on Faith’s phylogenetic diversity metric, indicated there was a statistically significant difference between at least two groups (*P* = 0.0039). Tukey’s multiple comparisons test found that the mean Faith’s PD alpha diversity for samples taken from the healthy phase (6.62 ± 1.45) was significantly lower than samples from the CKD (7.92 ± 1.23) phase (*P* adj. = 0.0219) and the post-transplant phase (8.076 ± 0.87), *P* adj. = 0.0052. No significant difference was found between the mean alpha diversity of the CKD phase (7.922) and the post-transplant phase using the Faith’s PD metric, *P* adj. = 0.9394.

### Beta diversity of treatment runs in healthy, CKD, and post-transplant phases

**Fig 7:**
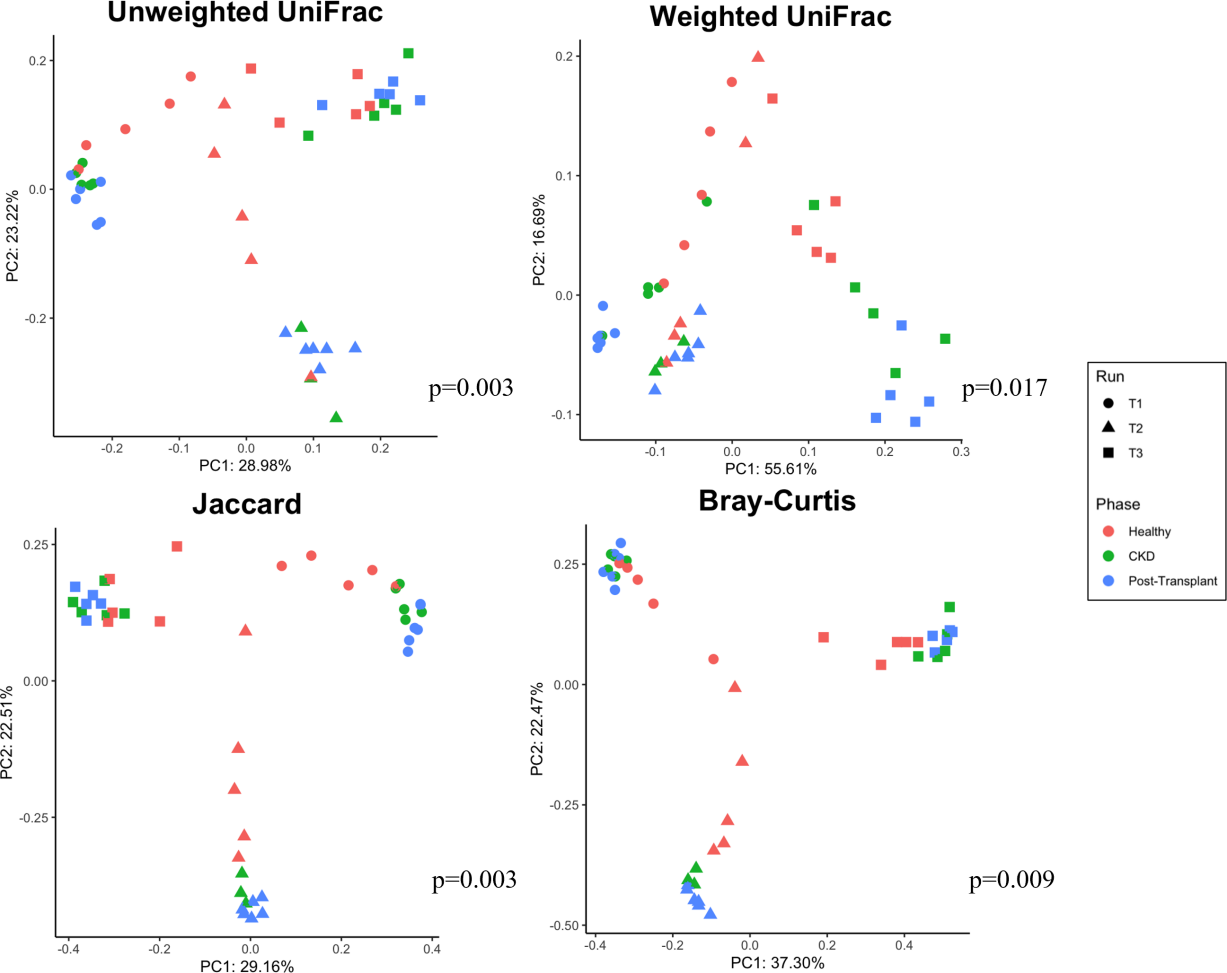
Beta diversity analysis of samples from treatment CDFF runs (T1, circle symbols; T2, triangle symbols; T3, square symbols) measured via four metrics (Jaccard, Bray-Curtis, unweighted UniFrac, and weighted UniFrac) for three phases of artificial saliva with urea treatment (healthy, CKD, and post-transplant) in red, green, and blue (respectively). Significance (*P*) relative to overall PERMANOVA testing between phases is indicated for each model.

Beta diversity was measured using the unweighted UniFrac, weighted UniFrac, Jaccard, and Bray-Curtis distance metrics. Following principal coordinates analysis, plotting (Fig. 7) showed clear distinct clustering based upon treatment run (shapes) as well as clustering based upon treatment phase (colors). Different clustering patterns based upon the treatment phase were confirmed statistically via permutational multivariate ANOVA tests (999 permutations), which found an overall significant difference between phases using every metric tested (unweighted UniFrac *P* = 0.003, weighted UniFrac *P* = 0.017, Jaccard *P* = 0.003, and Bray-Curtis *P* = 0.009).

Pairwise post hoc statistical comparisons found differences in beta diversity between healthy and CKD phases using three out of four metrics (unweighted UniFrac *P* adj. = 0.036, Jaccard *P* adj. = 0.033, and Bray-Curtis *P* adj. = 0.050). Comparing the healthy phase with the post-transplant phase, post hoc analysis revealed differences using all four metrics (unweighted UniFrac *P* adj. = 0.006, weighted UniFrac *P* adj. = 0.015, Jaccard *P* adj. = 0.006, and Bray-Curtis *P* adj. = 0.003). Comparisons of the CKD and post-transplant phases showed no significant differences using any beta diversity metric.

### Relative abundance of genera in treatment runs (T1, T2, and T3)

**Fig 8:**
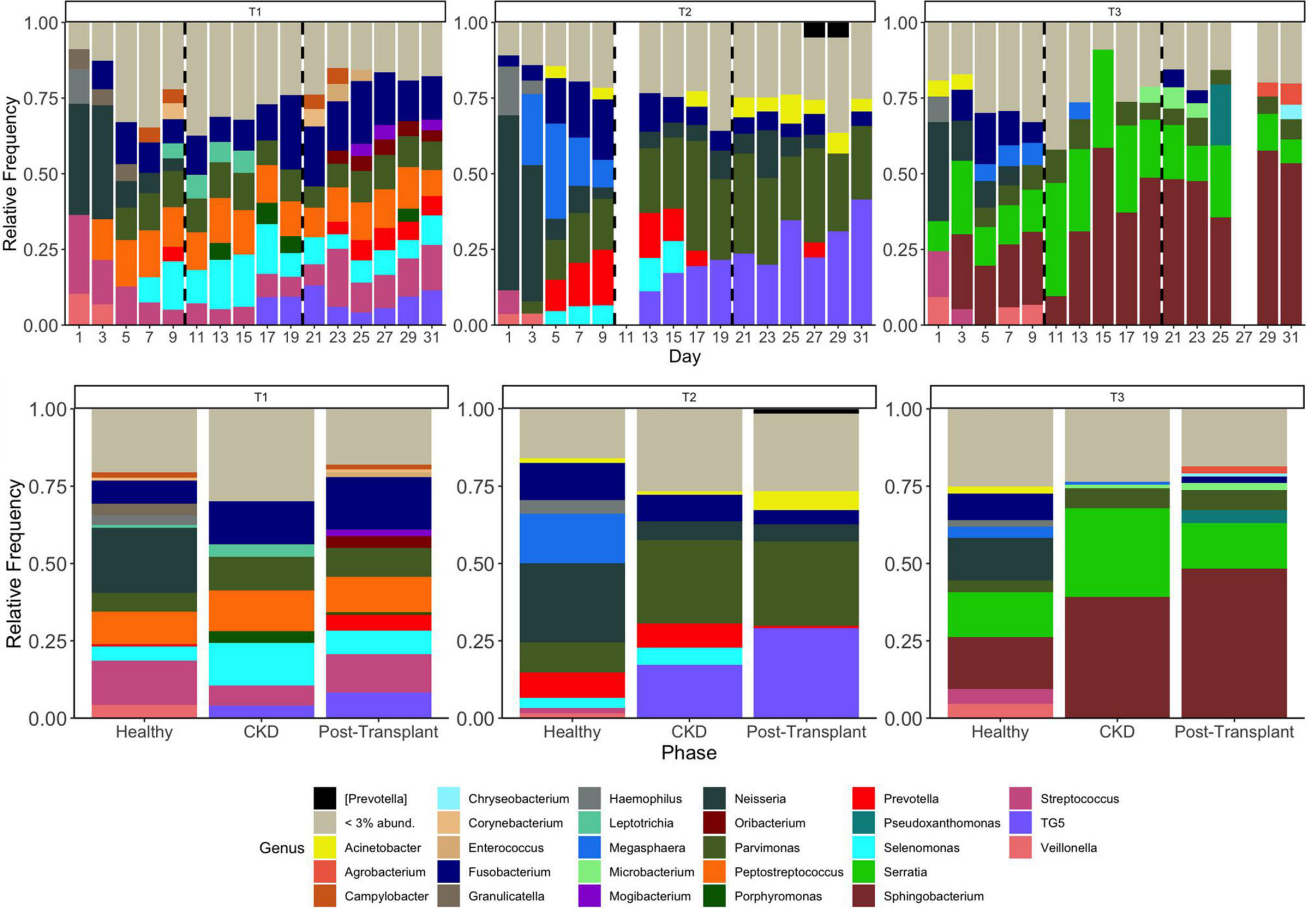
(Top) Microbiome profiles of oral microcosm samples collected from CDFF treatment runs (T1, T2, and T3). The stacked bar chart shows the mean proportion of genera (relative abundance) in each sample from each treatment run through healthy (days 1–10), CKD (days 11–20, between dashed lines), and post-transplant (days 21–31) phases. (Bottom) Mean relative abundance of genera from each treatment run in healthy (days 1–10), CKD (days 11–20), and post-transplant (days 21–31) phases. Each genus is represented by a different color (legend below). Genera found at relative abundance below 3% are grouped together (gray). *Prevotella* in square brackets denotes recommended, but not verified, taxonomies in the Greengenes database.

The three treatment runs established microbiome profiles with *Streptococcus*, *Neisseria*, *Veillonella,* and *Haemophilus* among the genera above 3% abundance in each run (Fig. 8). The 10 genera with the highest (sum) relative abundance are shown for each phase in Table 2 and include *Parvimonas*, *Sphingobacterium*, *Fusobacterium*, *Neisseria*, TG5, *Serratia*, *Streptococcus*, *Selenomonas*, *Prevotella,* and *Peptostreptococcus*. In treatment runs T1 and T2, gradual increases in TG5 are apparent from days 17 and 13 (respectively) (Fig. 8), and this is reflected in the overall abundance in each phase shown in Table 2. In treatment run T3, a gradual increase in *Sphingobacterium* is apparent (Fig. 8) and reflected in Table 2.

**TABLE 2:** The 10 defined genera with the highest combined relative abundance in treatment runs^*a*^

Genera	Healthy (%)	CKD (%)	Post-transplant (%)
*Parvimonas*	5.45	8.44	11.09
*Sphingobacterium*	3.63	9.86	10.70
*Fusobacterium*	7.43	5.81	6.98
*Neisseria*	15.10	1.32	1.61
TG5	0.18	4.03	9.91
*Serratia*	3.07	7.27	3.13
*Streptococcus*	5.53	2.28	3.73
*Selenomonas*	2.80	5.61	3.01
*Prevotella*	3.78	3.01	2.94
*Peptostreptococcus*	2.66	3.59	3.47

^*a*^ Relative abundance of each genus in the healthy (days 1–10), CDK (days 11–20), and post-transplant (days 21–31) phases is shown.

## DISCUSSION

Kidney failure progresses through CKD toward ESRD. In doing so, kidney function shows a continual decline and a reduced capacity to metabolize uremic toxins, such as creatinine and urea (14). Ultimately, renal replacement therapy in the form of dialysis or transplantation is required to preserve life. The inability to process uremic toxins causes their accumulation in the serum and saliva (14). In the saliva, median urea concentrations of CKD patients can rise up to 4.5 times higher than healthy controls (16). The oral microbiome is detectably different following kidney transplantation (1). Evidence from microbiome studies has suggested an increase in opportunistic pathogens following transplantation (2–5). Yet, these changes in oral bacterial communities have not been well studied. To investigate this, the present study used *in vitro* models of microcosm communities to study the response of oral bacteria to CKD and renal transplant.

The two (validation) runs, which were drip-fed McBain medium with the urea concentration of the saliva in healthy individuals (16) for 21 days, reached dynamic stability (measured by the congruence of viable counts) within 5 days, similar to previous studies (39, 40, 75, 76). Few examples exist that have employed the (highly sensitive) high-throughput sequencing method in CDFFs (75). The present study suggests that using this more sensitive technique, measurable changes might still occur (in the absence of a change in experimental conditions) beyond stable population sizes according to viable counts. Alpha and beta diversity showed significantly different patterns in early and late phases of validation runs, and trends, such as increasing relative abundance of genus TG5 and decreasing abundance of *Neisseria*, were apparent until the runs were terminated.

Three treatment runs were also performed to model the successive urea concentrations that the oral microbiome of a kidney transplant recipient might be exposed to. The healthy phase differed from both the CKD and post-transplant phases in terms of (Faith’s) alpha diversity and [(both): unweighted UniFrac, Jaccard, and Bray; (post-transplant only): weighted UniFrac] beta diversity metrics. Taxa found in (consensus) differential abundance between these phases included *V. dispar*, which has previously been found to outperform other oral species in CDFFs where urea has been removed after establishment (77) and TG5, which detectably increased in abundance in the absence of urea concentration change (Validations runs), and might represent ongoing ecological succession occurring independent of urea concentration change. Limited differences were observed in the comparison of the CKD and post-transplant model phases. No difference was observed in alpha or beta diversity, and only one consensus ASV was detected in differential abundance.

The limited differences observed in the oral microcosms during the treatment phases could reflect the fact that other factors drive the changes observed in patient studies or that the observation period for these models (11 days “post-transplant”) was too short. Future studies using CDFFs to model the effect of the onset and resolution of CKD on the oral microbiome could include replicate CDFFs inoculated with the same communities and apply variations in urea concentration in parallel. Such studies can establish more consistent baseline microbial population dynamics, including underlying growth rates and microbial interactions, facilitating a better understanding of oral microbiome changes that occur after transplantation (5).

## Data Availability

The data presented in the study are deposited in the NCBI SRA repository, accession number PRJNA1141113.

## References

[1] Campbell PM, Humphreys GJ, Summers AM, Konkel JE, Knight CG, Augustine T, McBain AJ. 2020. Does the microbiome affect the outcome of renal transplantation? Front Cell Infect Microbiol 10:558644. doi:10.3389/fcimb.2020.558644 33425774 PMC7785772

[2] Saraiva L, Lotufo RFM, Pustiglioni AN, Silva Jr HT, Imbronito AV. 2006. Evaluation of subgingival bacterial plaque changes and effects on periodontal tissues in patients with renal transplants under immunosuppressive therapy. Oral Surg Oral Med Oral Pathol Oral Radiol Endod 101:457–462. doi:10.1016/j.tripleo.2005.08.004 16545709

[3] Spolidorio LC, Spolidorio DMP, Massucato EMS, Neppelenbroek KH, Campanha NH, Sanches MH. 2006. Oral health in renal transplant recipients administered cyclosporin A or tacrolimus. Oral Dis 12:309–314. doi:10.1111/j.1601-0825.2005.01200.x 16700742

[4] Diaz PI, Hong B-Y, Frias-Lopez J, Dupuy AK, Angeloni M, Abusleme L, Terzi E, Ioannidou E, Strausbaugh LD, Dongari-Bagtzoglou A. 2013. Transplantation-associated long-term immunosuppression promotes oral colonization by potentially opportunistic pathogens without impacting other members of the salivary bacteriome. Clin Vaccine Immunol 20:920–930. doi:10.1128/CVI.00734-12 23616410 PMC3675961

[5] Fricke WF, Maddox C, Song Y, Bromberg JS. 2014. Human microbiota characterization in the course of renal transplantation. Am J Transplant 14:416–427. doi:10.1111/ajt.12588 24373208

[6] Cippà PE, Schiesser M, Ekberg H, van Gelder T, Mueller NJ, Cao CA, Fehr T, Bernasconi C. 2015. Risk stratification for rejection and infection after kidney transplantation. Clin J Am Soc Nephrol 10:2213–2220. doi:10.2215/CJN.01790215 26430088 PMC4670759

[7] Han YW, Wang X. 2013. Mobile microbiome: oral bacteria in extra-oral infections and inflammation. J Dent Res 92:485–491. doi:10.1177/0022034513487559 23625375 PMC3654760

[8] Hu J, Iragavarapu S, Nadkarni GN, Huang R, Erazo M, Bao X, Verghese D, Coca S, Ahmed MK, Peter I. 2018. Location-specific oral microbiome possesses features associated with CKD. Kidney Int Rep 3:193–204. doi:10.1016/j.ekir.2017.08.018 29340331 PMC5762954

[9] Simões-Silva L, Araujo R, Pestana M, Soares-Silva I, Sampaio-Maia B. 2018. The microbiome in chronic kidney disease patients undergoing hemodialysis and peritoneal dialysis. Pharmacol Res 130:143–151. doi:10.1016/j.phrs.2018.02.011 29444477

[10] Wallace MA. 1998. Anatomy and physiology of the kidney. AORN J 68:800. doi:10.1016/s0001-2092(06)62377-6 9829131

[11] Kulda V. 2012. Vitamin D metabolism. Vnitr Lek 58:400–404. 22716179

[12] Naber T, Purohit S. 2021. Chronic kidney disease: role of diet for a reduction in the severity of the disease. Nutrients 13:3277. doi:10.3390/nu13093277 34579153 PMC8467342

[13] Kato S, Chmielewski M, Honda H, Pecoits-Filho R, Matsuo S, Yuzawa Y, Tranaeus A, Stenvinkel P, Lindholm B. 2008. Aspects of immune dysfunction in end-stage renal disease. Clin J Am Soc Nephrol 3:1526–1533. doi:10.2215/CJN.00950208 18701615 PMC4571158

[14] Pandya D, Nagrajappa AK, Ravi KS. 2016. assessment and correlation of urea and creatinine levels in saliva and serum of patients with chronic kidney disease, diabetes and hypertension- a research study. J Clin Diagn Res 10:ZC58–ZC62. doi:10.7860/JCDR/2016/20294.8651 PMC5121806 27891460

[15] Rysz J, Gluba-Brzózka A, Franczyk B, Jabłonowski Z, Ciałkowska-Rysz A. 2017. Novel biomarkers in the diagnosis of chronic kidney disease and the prediction of its outcome. Int J Mol Sci 18:1702. doi:10.3390/ijms18081702 28777303 PMC5578092

[16] Lasisi TJ, Raji YR, Salako BL. 2016. Salivary creatinine and urea analysis in patients with chronic kidney disease: a case control study. BMC Nephrol 17:10. doi:10.1186/s12882-016-0222-x 26775026 PMC4715295

[17] Ratzke C, Gore J. 2018. Modifying and reacting to the environmental pH can drive bacterial interactions. PLoS Biol 16:e2004248. doi:10.1371/journal.pbio.2004248 29538378 PMC5868856

[18] Bowden GH, Hamilton IR. 1987. Environmental pH as a factor in the competition between strains of the oral streptococci Streptococcus mutans, S. sanguis, and "S. mitior" growing in continuous culture. Can J Microbiol 33:824–827. doi:10.1139/m87-143 3690424

[19] Quivey RG, Kuhnert WL, Hahn K. 2000. Adaptation of oral streptococci to low pH. Adv Microb Physiol 42:239–274. doi:10.1016/s0065-2911(00)42004-7 10907552

[20] Marsh PD, Devine DA. 2011. How is the development of dental biofilms influenced by the host? J Clin Periodontol 38:28–35. doi:10.1111/j.1600-051X.2010.01673.x 21323701

[21] Doel JJ, Benjamin N, Hector MP, Rogers M, Allaker RP. 2005. Evaluation of bacterial nitrate reduction in the human oral cavity. Eur J Oral Sci 113:14–19. doi:10.1111/j.1600-0722.2004.00184.x 15693824

[22] Lundberg JO, Weitzberg E, Gladwin MT. 2008. The nitrate–nitrite–nitric oxide pathway in physiology and therapeutics. Nat Rev Drug Discov 7:156–167. doi:10.1038/nrd2466 18167491

[23] Kapil V, Haydar SMA, Pearl V, Lundberg JO, Weitzberg E, Ahluwalia A. 2013. Physiological role for nitrate-reducing oral bacteria in blood pressure control. Free Radic Biol Med 55:93–100. doi:10.1016/j.freeradbiomed.2012.11.013 23183324 PMC3605573

[24] Cao X, Qian D, Meng X. 2013. Effects of pH on nitrite accumulation during wastewater denitrification. Environ Technol 34:45–51. doi:10.1080/09593330.2012.679700 23530314

[25] Rosier BT, Moya-Gonzalvez EM, Corell-Escuin P, Mira A. 2020. isolation and characterization of nitrate-reducing bacteria as potential probiotics for oral and systemic health. Front Microbiol 11:555465. doi:10.3389/fmicb.2020.555465 33042063 PMC7522554

[26] Rosier BT, Marsh PD, Mira A. 2018. Resilience of the oral microbiota in health: mechanisms that prevent dysbiosis. J Dent Res 97:371–380. doi:10.1177/0022034517742139 29195050

[27] Liu Y-L, Nascimento M, Burne RA. 2012. Progress toward understanding the contribution of alkali generation in dental biofilms to inhibition of dental caries. Int J Oral Sci 4:135–140. doi:10.1038/ijos.2012.54 22996271 PMC3465751

[28] Kleinberg I, Jenkins GN, Chatterjee R, Wijeyeweera L. 1982. The antimony pH electrode and its role in the assessment and interpretation of dental plaque pH. J Dent Res 61:1139–1147. doi:10.1177/00220345820610100601 6749924

[29] Burne RA, Marquis RE. 2000. Alkali production by oral bacteria and protection against dental caries. FEMS Microbiol Lett 193:1–6. doi:10.1111/j.1574-6968.2000.tb09393.x 11094270

[30] Andrade MRTC, Antunes LAA, Soares RM de A, Leão ATT, Maia LC, Primo LG. 2014. Lower dental caries prevalence associated to chronic kidney disease: a systematic review. Pediatr Nephrol 29:771–778. doi:10.1007/s00467-013-2437-4 23595424

[31] Peterson S, Woodhead J, Crall J. 1985. Caries resistance in children with chronic renal failure: plaque pH, salivary pH, and salivary composition. Pediatr Res 19:796–799. doi:10.1203/00006450-198508000-00003 3898000

[32] Evans RDR, Hemmila U, Mzinganjira H, Mtekateka M, Banda E, Sibale N, Kawale Z, Phiri C, Dreyer G, Calice-Silva V, Raimann JG, Levin N, Pecoits-Filho R, Mehta R, Macedo E. 2020. Diagnostic performance of a point-of-care saliva urea nitrogen dipstick to screen for kidney disease in low-resource settings where serum creatinine is unavailable. BMJ Glob Health 5:e002312. doi:10.1136/bmjgh-2020-002312 PMC7228485 32371573

[33] Lyman JL. 1986. Blood urea nitrogen and creatinine. Emerg Med Clin North Am 4:223–233. 3516645

[34] Lardner A. 2001. The effects of extracellular pH on immune function. J Leukoc Biol 69:522–530. doi:10.1189/jlb.69.4.522 11310837

[35] Erra Díaz F, Dantas E, Geffner J. 2018. Unravelling the interplay between extracellular acidosis and immune cells. Mediators Inflamm 2018:1218297. doi:10.1155/2018/1218297 30692870 PMC6332927

[36] Ledder RG, Mistry H, Sreenivasan PK, Humphreys G, McBain AJ. 2017. Arginine exposure decreases acidogenesis in long-term oral biofilm microcosms. mSphere 2:e00295-17. doi:10.1128/mSphere.00295-17 28861520 PMC5566835

[37] McBain AJ, Bartolo RG, Catrenich CE, Charbonneau D, Ledder RG, Gilbert P. 2003. Effects of a chlorhexidine gluconate-containing mouthwash on the vitality and antimicrobial susceptibility of in vitro oral bacterial ecosystems. Appl Environ Microbiol 69:4770–4776. doi:10.1128/AEM.69.8.4770-4776.2003 12902270 PMC169085

[38] McBain AJ, Bartolo RG, Catrenich CE, Charbonneau D, Ledder RG, Gilbert P. 2003. Effects of triclosan-containing rinse on the dynamics and antimicrobial susceptibility of in vitro plaque ecosystems. Antimicrob Agents Chemother 47:3531–3538. doi:10.1128/AAC.47.11.3531-3538.2003 14576113 PMC253811

[39] Ledder RG, Madhwani T, Sreenivasan PK, De Vizio W, McBain AJ. 2009. An in vitro evaluation of hydrolytic enzymes as dental plaque control agents. J Med Microbiol 58:482–491. doi:10.1099/jmm.0.006601-0 19273645

[40] McBain AJ, Bartolo RG, Catrenich CE, Charbonneau D, Ledder RG, Gilbert P. 2003. Growth and molecular characterization of dental plaque microcosms. J Appl Microbiol 94:655–664. doi:10.1046/j.1365-2672.2003.01876.x 12631201

[41] McBain AJ, Sissons C, Ledder RG, Sreenivasan PK, De Vizio W, Gilbert P. 2005. Development and characterization of a simple perfused oral microcosm. J Appl Microbiol 98:624–634. doi:10.1111/j.1365-2672.2004.02483.x 15715865

[42] Muras A, Mayer C, Otero-Casal P, Exterkate RAM, Brandt BW, Crielaard W, Otero A, Krom BP. 2020. Short-Chain N-acylhomoserine lactone quorum-sensing molecules promote periodontal pathogens in in vitro oral biofilms. Appl Environ Microbiol 86:e01941-19. doi:10.1128/AEM.01941-19 PMC6974637 31757829

[43] Soergel DAW, Dey N, Knight R, Brenner SE. 2012. Selection of primers for optimal taxonomic classification of environmental 16S rRNA gene sequences. ISME J 6:1440–1444. doi:10.1038/ismej.2011.208 22237546 PMC3379642

[44] D’Amore R, Ijaz UZ, Schirmer M, Kenny JG, Gregory R, Darby AC, Shakya M, Podar M, Quince C, Hall N. 2016. A comprehensive benchmarking study of protocols and sequencing platforms for 16S rRNA community profiling. BMC Genomics 17:55. doi:10.1186/s12864-015-2194-9 26763898 PMC4712552

[45] de Muinck EJ, Trosvik P, Gilfillan GD, Hov JR, Sundaram AYM. 2017. A novel ultra high-throughput 16S rRNA gene amplicon sequencing library preparation method for the Illumina HiSeq platform. Microbiome 5:68. doi:10.1186/s40168-017-0279-1 28683838 PMC5501495

[46] Bolyen E, Rideout JR, Dillon MR, Bokulich NA, Abnet CC, Al-Ghalith GA, Alexander H, Alm EJ, Arumugam M, Asnicar F, et al.. 2019. Reproducible, interactive, scalable and extensible microbiome data science using QIIME 2. Nat Biotechnol 37:852–857. doi:10.1038/s41587-019-0209-9 31341288 PMC7015180

[47] Callahan BJ, McMurdie PJ, Rosen MJ, Han AW, Johnson AJA, Holmes SP. 2016. DADA2: high-resolution sample inference from Illumina amplicon data. Nat Methods 13:581–583. doi:10.1038/nmeth.3869 27214047 PMC4927377

[48] Amir A, McDonald D, Navas-Molina JA, Kopylova E, Morton JT, Zech Xu Z, Kightley EP, Thompson LR, Hyde ER, Gonzalez A, Knight R. 2017. Deblur rapidly resolves single-nucleotide community sequence patterns. mSystems 2:e00191-16. doi:10.1128/mSystems.00191-16 28289731 PMC5340863

[49] Katoh K, Misawa K, Kuma K, Miyata T. 2002. MAFFT: a novel method for rapid multiple sequence alignment based on fast Fourier transform. Nucleic Acids Res 30:3059–3066. doi:10.1093/nar/gkf436 12136088 PMC135756

[50] Price MN, Dehal PS, Arkin AP. 2010. FastTree 2–approximately maximum-likelihood trees for large alignments. PLoS One 5:e9490. doi:10.1371/journal.pone.0009490 20224823 PMC2835736

[51] Shannon CE. 1948. A mathematical theory of communication. Bell Syst Tech J 27:379–423. doi:10.1002/j.1538-7305.1948.tb01338.x

[52] Pielou EC. 1975. Ecological diversity

[53] Faith DP. 1992. Conservation evaluation and phylogenetic diversity. Biol Conserv 61:1–10. doi:10.1016/0006-3207(92)91201-3

[54] Whittaker RH. 1960. Vegetation of the Siskiyou mountains, Oregon and California. Ecol Monogr 30:279–338. doi:10.2307/1943563

[55] Whittaker RH. 1972. Evolution and measurement of species diversity. Taxon 21:213–251. doi:10.2307/1218190

[56] Jaccard P. 1912. The distribution of the flora in the alpine zone.^1^. New Phytol 11:37–50. doi:10.1111/j.1469-8137.1912.tb05611.x

[57] Bray JR, Curtis JT. 1957. An ordination of the upland forest communities of southern Wisconsin. Ecol Monogr 27:325–349. doi:10.2307/1942268

[58] Lozupone C, Knight R. 2005. UniFrac: a new phylogenetic method for comparing microbial communities. Appl Environ Microbiol 71:8228–8235. doi:10.1128/AEM.71.12.8228-8235.2005 16332807 PMC1317376

[59] Lozupone CA, Hamady M, Kelley ST, Knight R. 2007. Quantitative and qualitative β diversity measures lead to different insights into factors that structure microbial communities. Appl Environ Microbiol 73:1576–1585. doi:10.1128/AEM.01996-06 17220268 PMC1828774

[60] Anderson MJ. 2001. A new method for non‐parametric multivariate analysis of variance. Austral Ecol 26:32–46. doi:10.1111/j.1442-9993.2001.01070.pp.x

[61] RStudio Team. 2020. RStudio: integrated development for R

[62] Bisanz JE. 2018. qiime2R: importing QIIME2 artifacts and associated data into R sessions. Version 099

[63] McMurdie PJ, Holmes S. 2013. phyloseq: an R package for reproducible interactive analysis and graphics of microbiome census data. PLoS One 8:e61217. doi:10.1371/journal.pone.0061217 23630581 PMC3632530

[64] Wickham H, Averick M, Bryan J, Chang W, McGowan L, François R, Grolemund G, Hayes A, Henry L, Hester J, Kuhn M, Pedersen T, Miller E, Bache S, Müller K, Ooms J, Robinson D, Seidel D, Spinu V, Takahashi K, Vaughan D, Wilke C, Woo K, Yutani H. 2019. Welcome to the Tidyverse. J Open Sour Software 4:1686. doi:10.21105/joss.01686

[65] Bokulich NA, Kaehler BD, Rideout JR, Dillon M, Bolyen E, Knight R, Huttley GA, Gregory Caporaso J. 2018. Optimizing taxonomic classification of marker-gene amplicon sequences with QIIME 2’s q2-feature-classifier plugin. Microbiome 6:90. doi:10.1186/s40168-018-0470-z 29773078 PMC5956843

[66] McDonald D, Price MN, Goodrich J, Nawrocki EP, DeSantis TZ, Probst A, Andersen GL, Knight R, Hugenholtz P. 2012. An improved Greengenes taxonomy with explicit ranks for ecological and evolutionary analyses of bacteria and archaea. ISME J 6:610–618. doi:10.1038/ismej.2011.139 22134646 PMC3280142

[67] Wickham H. 2016. ggplot2-elegant graphics for data analysis. Springer International Publishing, Cham, Switzerland.

[68] Nearing JT, Douglas GM, Hayes MG, MacDonald J, Desai DK, Allward N, Jones CMA, Wright RJ, Dhanani AS, Comeau AM, Langille MGI. 2022. Microbiome differential abundance methods produce different results across 38 datasets. Nat Commun 13:342. doi:10.1038/s41467-022-28034-z 35039521 PMC8763921

[69] Mandal S, Van Treuren W, White RA, Eggesbø M, Knight R, Peddada SD. 2015. Analysis of composition of microbiomes: a novel method for studying microbial composition. Microb Ecol Health Dis 26:27663. doi:10.3402/mehd.v26.27663 26028277 PMC4450248

[70] Gloor G. 2015. ALDEx2: ANOVA-like differential expression tool for compositional data, p 1–11. In ALDEX manual modular. Vol. 20.

[71] Fernandes AD, Macklaim JM, Linn TG, Reid G, Gloor GB. 2013. ANOVA-like differential expression (ALDEx) analysis for mixed population RNA-Seq. PLoS One 8:e67019. doi:10.1371/journal.pone.0067019 23843979 PMC3699591

[72] Fernandes AD, Reid JN, Macklaim JM, McMurrough TA, Edgell DR, Gloor GB. 2014. Unifying the analysis of high-throughput sequencing datasets: characterizing RNA-seq, 16S rRNA gene sequencing and selective growth experiments by compositional data analysis. Microbiome 2:15. doi:10.1186/2049-2618-2-15 24910773 PMC4030730

[73] Love MI, Huber W, Anders S. 2014. Moderated estimation of fold change and dispersion for RNA-seq data with DESeq2. Genome Biol 15:550. doi:10.1186/s13059-014-0550-8 25516281 PMC4302049

[74] Weiss S, Xu ZZ, Peddada S, Amir A, Bittinger K, Gonzalez A, Lozupone C, Zaneveld JR, Vázquez-Baeza Y, Birmingham A, Hyde ER, Knight R. 2017. Normalization and microbial differential abundance strategies depend upon data characteristics. Microbiome 5:27. doi:10.1186/s40168-017-0237-y 28253908 PMC5335496

[75] Humphreys GJ, McBain AJ. 2019. Antagonistic effects of Streptococcus and Lactobacillus probiotics in pharyngeal biofilms. Lett Appl Microbiol 68:303–312. doi:10.1111/lam.13133 30776138

[76] Humphreys GJ, McBain AJ. 2013. Continuous culture of sessile human oropharyngeal microbiotas. J Med Microbiol 62:906–916. doi:10.1099/jmm.0.055806-0 23449870

[77] Shu M, Browngardt CM, Chen Y-Y, Burne RA. 2003. Role of urease enzymes in stability of a 10-species oral biofilm consortium cultivated in a constant-depth film fermenter. Infect Immun 71:7188–7192. doi:10.1128/IAI.71.12.7188-7192.2003 14638814 PMC308945

